# Databases and Bioinformatic Tools for Glycobiology and Glycoproteomics

**DOI:** 10.3390/ijms21186727

**Published:** 2020-09-14

**Authors:** Xing Li, Zhijue Xu, Xiaokun Hong, Yan Zhang, Xia Zou

**Affiliations:** Key Laboratory of Systems Biomedicine (Ministry of Education), Shanghai Center for Systems Biomedicine, Shanghai Jiao Tong University, Shanghai 200240, China; xingli87@126.com (X.L.); xuzhijue@gmail.com (Z.X.); hongxk@sjtu.edu.cn (X.H.)

**Keywords:** glycosylation, glycoinformatics, glycan, glycoprotein, glycogene, lectin

## Abstract

Glycosylation plays critical roles in various biological processes and is closely related to diseases. Deciphering the glycocode in diverse cells and tissues offers opportunities to develop new disease biomarkers and more effective recombinant therapeutics. In the past few decades, with the development of glycobiology, glycomics, and glycoproteomics technologies, a large amount of glycoscience data has been generated. Subsequently, a number of glycobiology databases covering glycan structure, the glycosylation sites, the protein scaffolds, and related glycogenes have been developed to store, analyze, and integrate these data. However, these databases and tools are not well known or widely used by the public, including clinicians and other researchers who are not in the field of glycobiology, but are interested in glycoproteins. In this study, the representative databases of glycan structure, glycoprotein, glycan–protein interactions, glycogenes, and the newly developed bioinformatic tools and integrated portal for glycoproteomics are reviewed. We hope this overview could assist readers in searching for information on glycoproteins of interest, and promote further clinical application of glycobiology.

## 1. Introduction

Glycosylation is known as one of the most ubiquitous and important post-translational modifications (PTMs) in nature. It is estimated that more than 50% of mammal proteins and 90% of secreted and membrane proteins are glycosylated [1]. Glycosylation controls and defines a wide range of biological events, including cellular signaling, ligand–receptor interactions, cell–cell communication, pathogen–host recognition, immunological responses, and, consequently, it is involved in many human diseases, including inflammation and cancer [2]. Furthermore, the importance of glycosylation is highlighted by the fact that it can influence the therapeutic properties of many therapeutic proteins [3]. Therefore, deciphering the glycocode in diverse cells and tissues offers opportunities to develop new disease biomarkers and more effective recombinant therapeutics.

However, studies of the glycome are more complicated compared with those of the genome and proteome due to the diversity and branched nature of glycans. The complexity is further increased by the fact that glycan expression on a single protein is subject to both macroheterogeneity (variable occupancy of different glycosylation sites) and microheterogeneity (variable distribution of different glycans attached to a single glycosylation site) [4]. In addition, due to the different expression of glycogenes such as glycosyltransferase and glucosidase in different cells and tissues, the glycans attached to the same protein are cell-, tissue-, organism-, and physiological state-dependent. Furthermore, the same glycan structure on different proteins may have different functions. Therefore, the interpretation of the glycosylation sites, the attached glycan structure, the protein scaffolds, and related glycogenes are crucial for understanding the biological function of glycosylation.

In past decades, with improvements in analytical methods including high performance separation techniques and mass spectrometry (MS)-based analysis techniques [5,6], the qualitative and quantitative data of glycans, glycosites, glycopeptides, and glycoproteins have increased tremendously. In addition, extensive application of lectin chips, glycan microarrays, and glycogene chips greatly increased the data of glycan–protein interactions and the glycogenome [7,8,9]. Consequently, the development of databases and informatics tools to store, retrieve, integrate, and interpret these data is one of the most active fields in glycobiology.

The first publicly available glycan structural database, the Complex Carbohydrate Structural Database (CCSD)—also known as the CarbBank—was developed in the late 1980s [10,11]. Inspired by this work, more and more databases have been developed, such as the GLYCOSCIENCE.de database [12] and the KEGG (Kyoto Encyclopedia of Genes and Genomes) GLYCAN database [13]. In the early 2000s, the Consortium for Functional Glycomics (CFG) project began with a comprehensive database containing information on glycan structures, glycan-binding proteins (GBPs), glycosyltransferases, and glycan-related gene knockout mice [14], while the Japan Consortium for Glycobiology and Glycotechnology DataBase (JCGGDB) collected information on mass spectrometry analysis, glycogenes, and the affinity constants between lectins and pyridylaminated glycans [15,16,17]. In recent years, various glycoinformatic databases and tools have been developed. Of note, significant progress has been made lately through a new generation of centralized and integrated resources, including GlyGen [18], Glycomics@ExPASy [19], and GlyCosmos [20]. In addition to these comprehensive resources, a variety of databases on glycan structure, glycogenes, glycoproteins, and a series of bioinformatic tools based on these databases have been developed all over the world, which also promote the development of glycobiology. However, generally speaking, these databases and tools have not been well known and widely used by the public, including clinicians and other researchers who are not in the field of glycobiology, but are interested in glycoproteins.

**Figure 1:**
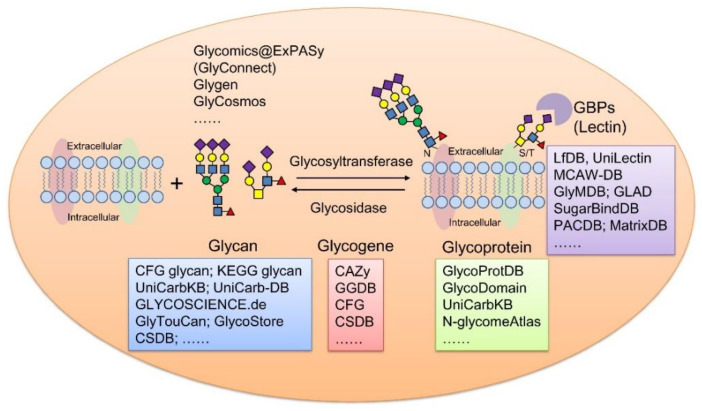
Summary of representative glycoinformatic databases using membrane glycoproteins as example. GBPs, glycan binding proteins.

As glycoproteins have become the promising candidates of disease markers and therapeutic targets, we mainly focus on providing an overview of main features and functionalities of the representative databases of glycan structure, glycoproteins, glycan–protein interactions, and glycogenes in humans and mammalians (Figure 1). In addition, we summarized some newly developed bioinformatic tools and integrated resources for glycoproteomics with the goal of making these bioinformatics resources more widely known to the public, especially to researchers in other disciplines. We hope this overview assists readers in searching for information on glycoproteins of interest, and promotes further clinical application of glycobiology.

## 2. Glycan Structure Databases

As different glycan structures have different regulatory effects on protein function, the analysis of glycan structure is one of the most important parts of glycobiology. According to the attached amino acid residues, glycans are divided into *N*-glycan, *O*-glycan, and other minor modifications. *N*-glycans are attached to the amino group of the Asn residue on the protein and can be released by endoglycosidases such as PNGaseF, while *O*-glycans are attached to the hydroxyl group of Ser or Thr and rarely on Tyr, and can be released by β-elimination. To determine the structure of a glycan, identification and quantification of monosaccharides, as well as identification of glycosidic bonds between monosaccharides, are both required. Useful information can be obtained by comparing the chromatography and mass spectra of unidentified glycans and known structures, which will accelerate the analysis [21,22,23,24]. For this purpose, storage and management of glycan structure data are very important. The first database in glycobiology, CarbBank, was constructed for collecting the glycan structures and the original mass spectra. There were more than 40,000 glycan structure entries in CarbBank until it was discontinued in 1997. These data are currently stored in multiple databases, including the CFG glycan databases, KEGG glycan, GLYCOSCIENCE.de, and CSDB (Carbohydrate Structure DataBase). Benefiting from the accumulating data of glycan structure, as well as the development of mass spectrometry technology, glycan structure identification is becoming more and more rapid, accurate, and automatic [23].

### 2.1. CFG Glycan Structure Database

The Consortium for Functional Glycomics (CFG, http://www.functionalglycomics.org/) is one of the largest resource platforms in glycomics [14]. It contains databases on glycan structure, glycosyltransferases, GBPs, and glycogene knockout mice, and also provides services such as glycogene microarrays and glycan arrays. The CFG Glycan Structure Database (http://www.functionalglycomics.org/glycomics/molecule/jsp/carbohydrate/carbMoleculeHome.jsp) offers detailed structural and chemical information for thousands of *N*- and *O*-glycans, including both synthetic glycans and glycans derived from human and mouse tissues and cell lines. Glycan structures stored in CFG can be searched by glycan name, composition, molecular weight, motifs, cell lines, or tissue samples. The search results show the glycan structure, molecular weight, composition, biological source and references, and provide links to relevant entries in CFG and external databases including a 3D modeling feature. Of note, CFG also provides carbohydrate standards for mass spectrometry and glycosyltransferases for synthesis of glycans, which has greatly improved the analysis of glycan structure. CFG has previously provided glycans profiling service using a variety of analytical methods such as MALDI–MS and ESI–MS. However, this service was closed after 2011 and was transferred to the Dell/Haslam biopolymer mass spectrometry laboratory at Imperial College, London.

### 2.2. Glycan Mass Spectral DataBase

JCGGDB is a meta-database involving 15 original databases including glycan profile data from mass spectral, lectin array data, glycoprotein data, glycogene data, glycoepitope data, and experiment support. Glycan Mass Spectral DataBase (GMDB, https://jcggdb.jp/rcmg/glycodb/Ms_ResultSearch) in JCGGDB is a multi-stage tandem mass spectral database, which stores MS^2^, MS^3^, and MS^4^ spectra of structurally-defined *N*-and *O*-linked glycans, and glycolipid glycans, as well as the partial structures of these glycans using a MALDI–QIT–TOF mass spectrometer [16]. *N*-glycans and glycolipid glycans are mostly tagged with 2-aminopyridine (PA), which can be used for fluorescence detection in HPLC. More importantly, this database also provides their protocol for MS^n^ spectra acquisition, which greatly helps users to compare and analyze their results correctly through spectral matching. In this database, the MS data of glycans can be easily searched by composition or the m/z value of the precursor ion. Although this database has not been updated, it will still be useful for some users’ data analysis.

### 2.3. UniCarbKB

UniCarbKB (http://unicarbkb.org/), composed of GlycoSuiteDB, GlycoBase, and EUROCarbDB, aims to construct an information storage and search platform [25,26,27]. GlycoSuiteDB collects all the glycan structures and glycosylation site information published from 1990 to 2005. In this database, glycan structures can be searched by taxonomy, tissue, protein, and disease. Entries in GlycoSuiteDB contain the glycan structure, the glycosylation site on the protein, the biological source and literature references, and information on diseases [28,29]. In addition, UniCarbKB includes more than 350 glycan structures analyzed by HPLC previously available from GlycoBase [30], and MS, NMR, and HPLC experimental data for glycans from EUROCarbDB [31]. UniCarbKB also provides a series of bioinformatic tools for glycan analysis. For example, GlycoDigest can simulate the digestion of oligosaccharides by exoglycosydases [32], and UniCorn is a theoretical *N*-glycan structure database [21], which could be used to improve the efficiency of glycan analysis and validate glycan structures. UniCarbKB provides useful information on site-specific *N*- and *O*-glycans of glycoprotein, and connects glycan structure with its attached protein(s) which can be annotated in UniProtKB. Unfortunately, UniCarbKB cannot be accessed currently, possibly due to technical problems. As UniCarbKB stores valuable data for glycobiology studies, we hope this database can be functional again.

### 2.4. KEGG Glycan

KEGG is an integrated database containing the knowledge of molecular networks such as signaling and metabolic pathways. KEGG glycan (http://www.genome.jp/kegg/glycan/) collects the experimentally determined glycan structures, along with the glycan biosynthesis and metabolism pathways [13]. The glycan structures stored in KEGG glycan could be searched through the G number as well as the DBGET tool. Each entry includes the molecular weight, composition, detailed structure of the glycan, and the references. KEGG glycan also provides external links to other glycan databases for cross reference. In addition, glycan-related pathways and diseases are also important information provided by KEGG. The glycan-related pathways include biosynthesis and degradation of glycans, glycan-involved signaling molecules and interaction pathways, and cancer-associated carbohydrates, which could help researchers understand the biological role of glycans.

### 2.5. GLYCOSCIENCE.de

GLYCOSCIENCE.de (http://www.glycosciences.de/) is an integrated portal containing a combination of databases and tools for glycome analysis. Its main focus is on glycan 3D structures, which are extracted from CarbBank, Protein Data Bank (PDB), and literature research [12,33]. In addition to standard molecular information, GLYCOSCIENCES.de provides many useful tools for performing various quality checks and structural analyses of 3D structure modeling, detection, and validation of carbohydrates in PDB files, and supports MS/NMR analyses of glycans. For example, Sweet-II is a powerful tool in the GLYCOSCIENCE.de database, which can rapidly convert the sequence of a complex glycan into a reliable 3D molecular model for molecular dynamic simulations and other further analyses [34].

Glycosciences.DB (http://www.glycosciences.de/database/) is the main glycan structure database of GLYCOSCIENCE.de, collecting various kinds of data on glycan structures linking glycomics and proteomics data [35]. Glycosciences.DB provides three entries, including a glycan structure entry, literature entry, and worldwide Protein Data Bank (wwPDB) entry, all of which are linked to each other. Glycan structure can be searched by glycan (sub-)structure, monosaccharide composition, molecular formula, structure classification and motifs, as well as NMR, MS, PDB query, or bibliography queries. The graphical interface GlycanBuilder is also supported for database search. When users search specific glycan, the detailed information including glycan composition, glycan structure, NMR data, PDB link (if available), and references are provided. As the PDB database has not provided a way to search 3D structures of glycans, this database provides the only way to search for specific carbohydrate structures in PDB. In addition, more and more literature has been added to Glycosciences.DB, which increases the number of new glycan structure entries in this database. At the time of this writing, Glycosciences.DB contains more than 26,000 glycan structure entries with 13,500 3D structure models, which makes it one of the largest databases for glycan 3D structure analysis.

### 2.6. UniCarb-DB

UniCarb-DB (https://unicarb-db.expasy.org/) is an emerging public database providing access to a collection of LC–MS/MS glycan fragments released from glycoproteins for glycomic discovery [36]. UniCarb-DB provides a user-friendly search interface. Users can search glycan data by taxonomy, tissue, reference, mass, composition, or precursor mass using basic search or advanced search. The result shows detailed information, such as glycan types, linkages, structures, and the corresponding MS data obtained from the literature and experimental evidence. The links to PubMed and Uniprot entries are also presented. By comparing with the experimental spectra in the database, UniCarb-DB can be used as reference to aid manual annotation of glycan structure. UniCarb-DB contains over 1500 spectra of both *N*- and *O*-glycans of glycoproteins derived from 13 taxonomies and 20 tissues, and has grown to be one of the largest experimental glycomic MS databases.

### 2.7. GlyTouCan

GlyTouCan (https://glytoucan.org/) is an international glycan structure repository, which was developed on the basis of GlycomeDB [37]. GlycomeDB is established for integrating glycan structure information from different sources (CFG, BCSDB, GLYCOSCIENCES.de, KEGG glycan, EUROCarbDB, and CarbBank) and excluding the redundant parts [38,39]. Glycan structures in GlyTouCan can be searched by text input, motif, or drawing glycan structures in GlycanBuilder. Each entry shows the glycan structure, molecular weight, biological source, references and external links to other databases. The most important feature of GlyTouCan is that each glycan structure is assigned a globally unique accession number. This unique accession number, like the mRNA and protein accessions, can be cited when referring to a specific glycan, and will simplify and unify the description of glycans in scientific literature, making it possible to search between different databases. In addition, the developers of GlyTouCan encourage users to register identified glycans and obtain unique accessions for publication, which is similar to GeneBank [40].

### 2.8. GlycoStore

GlycoStore (https://www.glycostore.org), developed based on the publicly available experimental datasets GlycoBase, is an annotated database of retention properties of *N*-, *O*-, glycosphingolipid (GSL) glycans and free oligosaccharides of glycoproteins, glycolipids, and biotherapeutics [41]. GlycoStore focuses on ultra-high performance liquid chromatography (U/HPLC), reversed phase (RP)-U/HPLC, porous graphitized carbon (PGC) chromatography, and capillary electrophoresis (CE) elution positions for approximately 850 unique glycan structures with links to taxonomy, glycoprotein, and supporting literature. Data stored in this database can be searched by experimental values (GU, AU, or time), monosaccharide composition or metadata labels (taxonomy, sample name, and the Oxford linear notation). Each glycan structure entry page lists all methods for determining this structure and substances in which this glycan is present. Interestingly, GlycoStore provides a comparison function. Users can choose two to three proteins of interest, and the results will show common structures of these proteins.

### 2.9. CSDB

Carbohydrate Structure DataBase (CSDB, http://csdb.glycoscience.ru/database/) is the largest database focusing on the structures of glycans and glycoconjugates in prokaryotes, plants, and fungi. The current version of CSDB is a merger of the Bacterial (BCSDB) and Plant and Fungal (PFCSDB) databases [42]. CSDB contains manually curated structural, taxonomic, and NMR data of carbohydrates. CSDB has a high coverage of natural carbohydrates in bacteria, archaea, fungi, and plants, and is still being updated [43]. Users can search the database by IDs, bibliographic data and keywords, biological source, structural fragments, and NMR data, and also browse all the identified carbohydrates in a species. The results show the glycan structural data with an extended bibliography, assigned NMR spectra, taxon identification, and other information. Of note, CSDB provides high data quality by manual curation of original publications. In addition, CSDB also provides bioinformatic tools to interpret the carbohydrate structures from NMR spectra and predict it according to carbohydrate structures [44], as well as a number of computational services, such as NMR simulation and taxon clustering.

## 3. Glycoprotein Databases

*N*-and *O*-glycosylation are the most common glycosylation modifications on proteins. *N*-glycans are attached to the amino group of Asn residue on protein, with a consensus motif as Asn–Xaa–Ser/Thr, where Xaa could be any amino acid except Pro. *O*-glycosylation includes *O*-GalNAc, *O*-GlcNAc, *O*-Man, *O*-Fuc, and other types, in which *O*-GalNAc glycans initiated by *N*-acetyl-α-d-galactosamine (GalNAc) attached to the hydroxyl group of Ser or Thr residues is the most abundant type [45]. In addition, *O*-GlcNAcylation is a monosaccharide *N*-acetyl-β-d-glucosamine (GlcNAc) modification on nuclear, cytoplasmic, and mitochondrial proteins [46], which is different from other glycosylation types with complex glycans mainly attached to secreted and membrane proteins.

In order to identify glycoproteins, the glycosylated proteins should be enriched with analytical, affinity, or chemical techniques. A variety of technologies such as hydrophilic chromatography, hydrazide chemistry, lectin chromatography, or metabolic labeling have been widely used [17,47,48,49,50,51]. Sequentially, the complex glycans are removed or shortened by glycosidase treatment or chemical approaches and usually a unique tag is left or labeled at the glycosylation site. The peptides are then applied to mass spectrometry for identification. To identify the glycopeptides and glycosylation sites, different strategies are applied for different types of glycosylation. As the release of *N*-glycans by endoglycosidase facilitates high-throughput and automated identification for *N*-glycoproteins, a variety of *N*-glycosylation sites have been annotated. Based on these data, the Technical University of Denmark developed the NetNGlyc server to predict the *N*-glycosylation sites according to the well-known Asn–Xaa–Ser/Thr sequon. However, the identification of *O*-glycoproteins such as *O*-GalNAc and *O*-Man has lagged because of a lack of efficient glycosidases to release *O*-glycans. Recently, genetic technology named SimpleCell was developed to simplify the structure of *O*-glycans to a single GalNAc for lectin affinity enrichment and tag for glycosite identification by MS [52,53,54]. As a result, a large number of *O*-glycoproteins were identified, which greatly expanded the *O*-glycoprotein data pool. Here we describe the represented databases for *N*-glycoprotein and *O*-glycoprotein.

### 3.1. GlycoProtDB (GPDB)

GlycoProtDB (https://acgg.asia/gpdb2) is a database containing curated experimental data of *N*-glycoproteins. The *N*-glycosylation sites were identified through lectin affinity column combined with isotope-coded glycosylation site-specific tagging (IGOT) method. In detail, the proteins were digested and the corresponding *N*-glycosylated peptides were enriched by ConA or WGA lectin column. PNGaseF treatments were performed in a solvent using stable isotope-labeled water, H_2_^18^O, to remove the *N*-glycan and label the glycosylated Asn residue with ^18^O. The *N*-glycosylation sites were identified by LC–MS/MS [55]. This method was first applied to the model organism Caenorhabditis elegans and identified 250 glycoproteins carrying 400 unique *N*-glycosylation sites [17]. The current version of GlycoProtDB contains *N*-glycoproteins from C. elegans, different mouse tissues (C57BL/6, male) [56,57], and human cell lines [58]. Proteins in GlycoProtDB can be searched by gene symbol, gene name, or protein name. The results show a map of *N*-glycosylation sites on the protein, the amino acid sequence annotated with potential and identified *N*-glycosylation sites. Of note, protein sequences with common glycopeptide sequence(s) are linked each other, which may be convenient for users to analyze the glycosylation of homologous proteins.

### 3.2. UniPep and N-GlycositeAtlas

Unlike GlycoProtDB, UniPep (http://www.unipep.org/) is a database that focuses only on the human *N*-glycoprotein for glycoprotein biomarker discovery [59]. *N*-glycoproteins from plasma, bladder, breast cancer cells, liver, lymphocytes, cerebrospinal fluid, prostate tissue, and prostate cancer cells were captured using the hydrazide chemistry method, released by specific glycosidase PNGaseF, and identified by MS/MS [49]. The 1552 unique *N*-linked glycosylation sites were identified and mapped on the associated proteins, and then imported into the UniPep database [59]. The *N*-glycoproteins can be searched by gene symbol, gene name, Swiss-Prot ID, IPI ID, amino acid sequence, or peptide mass. In addition, users can also browse the identified list and the metabolic and signaling pathways for *N*-glycoproteins. Each *N*-glycoprotein is provided with detailed information including tables listing predicted and identified *N*-linked glycopeptides, protein sequence annotated with *N*-glycopeptides, and a map of *N*-glycosylation sites on the protein.

Recently, a larger database called *N*-GlycositeAtlas (http://nglycositeatlas.biomarkercenter.org) containing more than 30,000 glycosite-containing peptides with >14,000 *N*-glycosylation sites from over 7200 *N*-glycoproteins was developed by the same lab [22]. These human glycosite-containing peptides were collected from over 100 publications and unpublished datasets, and then mapped to UniProt database. Users can perform basic search or advanced search by gene/protein name, accession number, glycosylation site location, glycosite containing peptide, tissue/liquid/cell line, or publication. Overall, the most important feature of these two databases is that lots of *N*-glycoproteins were derived from clinical samples, including plasma, human-derived tissues, body fluids, and cell lines, which may be of interest to clinical researchers.

### 3.3. O-GalNAc Protein Databases

The GlycoDomain Viewer (https://glycodomain.glycomics.ku.dk/) established by the Copenhagen Center for Glycomics to organize and share the *O*-GalNAc proteome identified by SimpleCell technology [53], has been considered as one of the largest databases of *O*-GalNAc glycoprotein. Presently, this database includes 629 experimentally identified *O*-GalNAc glycoproteins and 2942 *O*-glycosylation sites from human and animal cell lines. It allows users to search by the NCBI gene name and the UniProt ID, or browse all the identified list. Each *O*-GalNAc glycoprotein in this dataset is shown with its sequence and domain topology. Importantly, the verified and predicted glycosylated sites of *N*-glycan, *O*-GalNAc, *O*-Mannose and *O*-Xylose are mapped on the protein sequence. By showing data produced experimentally as well as retrieved from other databases, GlycoDomain Viewer presents the interplay between relevant protein and post-translational modification information to explore the possible effects of glycosylation on a protein. In addition, based on this dataset, NetOGlyc was upgraded to version 4.0 (www.cbs.dtu.dk/services/NetOGlyc/), which could provide more accurate predictions for *O*-GalNAc glycosylation sites in mammalian proteins [53].

### 3.4. O-GlcNAc Protein Database

YinOYang 1.2 (http://www.cbs.dtu.dk/services/YinOYang/) is a prediction database for identifying potential *O*-GlcNAcylation sites for any submitted protein [60]. This server has incorporated results from NetPhos (http://www.cbs.dtu.dk/services/NetPhos/) [61]. The Ser/Thr residues which are predicted to be *O*-GlcNAcylated, as well as phosphorylated, are marked. Such sites may be reversibly and dynamically modified by *O*-GlcNAc or phosphate groups at different times in the cell. However, in comparison with *O*-GalNAc glycosylation, there is still a lack of a comprehensive available *O*-GlcNAc protein database.

## 4. Glycogene Databases

Different from nucleic acids and proteins, the synthesis of glycans is not template-driven. Instead, the glycosyltransferases control the structures of glycans and their attached sites on the carrier proteins, and the glycosidases further modify the glycan structures. These glycan-related genes are named glycogenes. There are about 200 glycogenes in humans, which cover about 1% of the human genome [15]. These glycogenes are expressed in a tissue- and time-specific pattern. The glycome of a specific tissue at a specific time point is determined by the combination of all present glycogenes. To understand the regulation of glycans, information about glycogenes should be collected. In this section, we describe several representative databases of glycogenes.

### 4.1. CAZy

The Carbohydrate-Active Enzymes database (CAZy, http://www.cazy.org/), the largest database of glycan-related genes, has collected enzymes that degrade, modify, or form glycosidic bonds and proteins containing carbohydrate-binding modules since 1998 [62]. Enzymes in CAZy are divided into five categories according to sequence and structure similarity including glycoside hydrolases (GHs), glycosyltransferases (GTs), polysaccharide lyases (PLs), carbohydrate esterases (CEs), and auxiliary activities (AAs). Users can search enzymes by protein name, organism name, GeneBank or UniProt accession, and EC number. The results contain the genomic, structural, and biochemical information on glyco-enzymes, as well as external links to GenBank, UniProt, CFG, and PDB. Application of this information can significantly facilitate the synthesis of biologically active glycan products. However, it is noteworthy that although CAZy stores information on several hundred thousands of enzymes from multiple taxa, but less than 5% of them have experimentally established activities [23].

To create the most reliable encyclopedia of carbohydrate-active enzymes possible, CAZy provides a community-driven resource named CAZypedia (http://www.cazypedia.org) [63]. Currently, the database contains a series of curator-approved content of glyco-enzymes, which can help researchers who are not in this field understand the basic knowledge of glycobiology. In addition, just like a wiki, the search and display pages of CAZypedia are very simple and very friendly for the beginners.

### 4.2. GGDB

The GlycoGene DataBase (GGDB, http://acgg.asia/ggdb2/), established by JCGGDB, includes identified genes related to glycan synthesis, such as glycosyltransferases, sugar nucleotide synthases, and sugar–nucleotide transporters [15]. As some proteoglycans such as heparan sulfate and chondroitin sulfate contain sulfo groups, a category of 34 sulfotransferases is also included in GGDB. Users can search by gene symbol or designation. Each gene provides mRNA and protein sequences, chromosome location, EC number, and gene ontology, as well as links to GeneBank, CAZy, and OMIM. References with brief annotations are also provided. In comparison with CAZy, GGDB provides more detailed information, such as donor and acceptor substrates, as well as the expression pattern for each gene. Moreover, researchers can order the plasmid conveniently according to the biological resource of each gene. GGDB also provides information on the tissue expression and a link to the glycogene knock-out mice resource if available, which can be very helpful for the functional study of glycogenes.

### 4.3. CFG Glycosyltransferases Database

The glycan structures, glycosyltransferases, and GBPs are the three main contents of the CFG databases. The CFG Glycosyltransferases database (http://www.functionalglycomics.org/glycomics/molecule/jsp/glycoEnzyme/geMolecule.jsp) provides a user-friendly graphical interface showing the structure of different glycans. By clicking a monosaccharide, users are directed to the information of the glycosyltransferase which forms this structure. General information such as enzyme name, EC number, organism, and detailed information such as nucleotide accession in GeneBank, expression profile, Swiss-Prot ID, amino acid sequence, and biochemical reaction are provided. There are also external links to PubMed, KEGG pathway, CAZy, and Swiss-Prot for cross reference. CFG also offers several types of resources, including glycogene microarray for determining glycogene expression profiles, glycotransferases, and reagents for studying the catalytic activity of glycotransferases, antibodies for histochemical staining and purification of glycoconjugates, synthetic glycans for studying the glycan–protein interactions, and glycogene knock-out mice for characterizing the biological role of glycogenes.

### 4.4. CSDB_GT Subdatabase

CSDB is a database of the glycan and glycoconjugate structures in prokaryotes, plants, and fungi. In 2017, CSDB established a curated database on carbohydrate-active enzymes called CSDB_GT (http://csdb.glycoscience.ru/gt.html). Currently, CSDB_GT contains glycosyltransferases found in Arabidopsis thaliana, Escherichia coli [64] and recently was expanded to Saccharomyces cerevisiae. Users can search for glycosyltransferases using the name or protein/gene database ID, type of glycan the enzyme takes part in, glycosidic bond the enzyme synthesizes, and the enzyme’s donor or acceptor. The results provide the name and links to NCBI and UniProt. Moreover, CSDB_GT provides information on carbohydrate structures and enzyme activity, which are supported by different levels of evidence that can be traced to original publications.

### 4.5. GlyMAP

Mutations in most glycogenes could cause a global defect of glycosylation. While some glycosyltransferase families are composed of homologous isoenzymes, mutations on one member may not affect glycosylation globally. Large-scale whole exome sequencing (WES) could provide information on mutations in glycosyltransferase genes in populations, and could be useful to analyze and predict the functional relationship between the glycogenes and diseases. From WES of 2000 Danes, Hansen et al. constructed a database of Functional Mutational Map of glycogenes (GlyMAP, http://glymap.glycomics.ku.dk/) [65] to provide the global map of glycogenome genetic stability. All missense mutations were collected in this database and deleterious mutation maps were drawn by prediction algorithms, manual inspection, and additional experimental analysis in CAZy family GT27. From these data, mutations with unknown functions could be related to specific disorders and may help in the discovery of novel congenital disorders of glycosylation (CDG).

## 5. Glycan-Protein Interaction Databases

The specific interactions between glycans and glycan binding proteins is an important part of the biological function of glycans. In recent years, because of the development of high-throughput technologies such as lectin arrays and glycan arrays, glycan–protein interaction data is growing rapidly. Several databases have been established to store and share the glycan–protein interaction data. In this section, we introduce some representative databases closely related to mammalians and human health.

### 5.1. LfDB

Lectins is one of the major categories of GBPs, which can selectively interact with glycans. The Lectin Frontier Database (LfDB, http://acgg.asia/lfdb2/), belonging to JCGGDB, is a database established to describe the quantitative interaction data between a number of lectins and various glycans. Users can simply search by keywords or choose categories among lectin family, monosaccharide specificity, or 3D-fold. The results are provided on two pages: the lectin information page including monosaccharide specificity, source of lectin and 3D structure if available; the interaction page displaying the affinity constants (Ka) of lectins toward a panel of glycans obtained by an automated frontal affinity chromatography system [66]. In the future, other GBPs such as anti-glycan antibodies will also be included in LfDB [67]. Information from LfDB could help researchers understand the structural basis of lectin–glycan interactions.

### 5.2. UniLectin

The recently released UniLectin (https://www.unilectin.eu/) is a new platform designed to cover the knowledge of lectins, their classification, and their biological role [68]. Currently, the platform consists of two modules UniLectin3D and PropLec. UniLectin3D, the main module of UniLectin, is a curated database of lectin 3D structures and interacting ligands. User can search lectins by keyword, kingdom order, historical classification, monosaccharide, associate IUPAC sequence, fold of the binding site, or multiple criteria. For each lectin, a detailed page with 3D visualization, interactions, and links to external databases is displayed. Another module, PropLec, is focused on β-propeller lectin prediction in all species. A quick search can be performed by keyword, accession number, species name, or protein name. Currently, UniLectin has exceeded 2000 lectin structures, which will make it an important tool in glycobiology research.

### 5.3. PACDB

The interaction between pathogen and host is mediated by cell surface molecules, such as proteins and glycolipids. The Pathogen Adherence to Carbohydrate Database (PACDB, https://acgg.asia/db/diseases/pacdb) is a database that was established to collect the information on pathogens (e.g., bacteria, fungus, toxin and virus) adhering to glycan expressed on the cell surface of host animals or plants [69]. Currently, there are more than 1800 interactions related to over 180 diseases in PACDB. Users can browse the database by pathogen names or disease names. Interaction information extracted from references are listed on the page of each pathogen or disease, and annotated with [binding] or [not binding]. The glycans can be linked to the JCGGDB database and the references are linked to PubMed for further interest. Although some interactions require confirmation, it provides a lead for further investigation of correlations between pathogen and host cells. Currently, the data of PACDB has been summarized in the GlyCosmos.

### 5.4. SugarBindDB

The SugarBind Database (SugarBindDB, https://sugarbind.expasy.org/) is another curated resource, covering knowledge of glycan-mediated host–pathogen interactions based on glycan–protein binding pairs [70]. A set of five inseparable components including the pathogenic agent, lectin adhesin, glycan ligand, disease, and references constitutes the core information. Users can search the database by several terms, such as pathogenic agents (such as influenza virus), ligands (such as A Lewis b), recognizing lectins or adhesins (BabA), affected area in the pathology (e.g., intestine), references, diseases, and multi–criteria. The database can also be queried by a glycan composition or a glycan structure drawn by GlycanBuilder. Each entry lists all related information with as much precision as possible in the form of graphs and text. Similar to PACDB, SugarBindDB provide literature for each glycan–protein binding pair. Moreover, this database also provides 3D structure from PDB and offers external links to protein and glycan-related resources such as UniProtKB, UniCarbKB, and CFG. As accumulating evidences show that glycans play important roles in the recognition between pathogen and host which is crucial to the entry and release of pathogen, SugarBindDB is therefore a valuable tool for mechanism study of pathogen infection and the toxicity of glycan-binding toxins.

### 5.5. GLAD: Glycan Array Dashboard

The glycan array is a high-throughput tool for profiling protein–glycan interactions. So far, thousands of glycan array experiments have been performed and a huge amount of data have been collected. However, the glycan array results were mostly stored as excel files (for example the CFG Core H glycan array data), which is not convenient to present the protein–glycan interactions visually. To address this limitation, GLAD (GLycan Array Dashboard, https://glycotoolkit.com/Tools/GLAD/) is a web-based tool developed to visualize, analyze, and present glycan array data [71]. Users input data as tab-delimited text files in the correct format, and then can visualize and select data for display using various types of charts, including grouped bar charts, heatmaps, and interaction networks. GLAD also allows users to filter, sort and normalize data to accentuate key data and binding relationships. Overall, GLAD is a useful tool to uncover hidden relationships between glycan array datasets.

### 5.6. MCAW-DB

Glycan recognition patterns are often obtained by glycan microarrays. Multiple Carbohydrate Alignment with Weights Database (MCAW-DB, https://mcawdb.glycoinfo.org/) is a glycan profiling database containing the multiple alignment analysis results of 1081 glycan microarray samples collected from the CFG to find the glycan substructures having higher binding affinity to GBPs [72]. On the search page, users can filter taxa, protein family, investigator and array version. Text search is also permitted. The results show detailed sample information, multiple alignment analysis results and data set details, as well being as linked to the CFG database.

### 5.7. GlyMDB

Unlike MCAW-DB only focuses on glycan sequence alignment, the Glycan Microarray DataBase (GlyMDB, http://www.glycanstructure.org/glymdb) is a comprehensive glycan microarray database and analysis tool for data visualization, binder/non-binder classification, glycan-binding motif discovery, and glycan array sample comparison [73]. There are two options on the main pages. On one hand, users can upload microarray spreadsheet files. On the other hand, a query can be made by protein name, protein sequence or PDB ID within the 5203 glycan microarray samples collected from the CFG in the database. The GlyMDB results show glycan ligand information, fluorescence intensity, and common motifs. In addition, this database also provides some commonly used tools, such as binder/non-binder classification, glycan-binding motif discovery, glycan array sample comparison, and cross-linking of the glycan microarray to PDB. In addition, GlyMDB also provides bioinformatic tools such as Glycan Reader and Glycan Modeler for visualization and simulation of glycan 3D structures.

### 5.8. MatrixDB

Glycoproteins, proteoglycans and glycosaminoglycans (GAGs) are highly enriched in the extracellular matrix (ECM). MatrixDB (http://matrixdb.univ-lyon1.fr/) is a freely available database focused on these extracellular interactions [74,75,76,77]. The interaction data in the MatrixDB is collected from experiments and literatures with curation, as well as adopted from the International Molecular Exchange consortium (IMEx) databases. As many extracellular proteins usually assemble into complex multimers, MatrixDB provides not only the interactions with individual polypeptides, but also the interactions with multimers, which provide a more comprehensive perspective to understand the extracellular interaction network. In addition, the interacting proteins are linked to their expression and localization data in multiple databases including UniGene, the Human Protein Atlas, and Expression Atlas, which enable the users to create tissue- and cell-specific interaction networks.

## 6. Software Tools for Glycan and Intact Glycopeptide Analysis

Mass spectrometry (MS) has long been considered as one of the most powerful techniques for glycomic and glycoproteomic study. In general, there are two main strategies for elucidating glycosylation information using MS techniques [24]. The first strategy is for global analysis of glycan structure released from glycoproteins using endoglycosidases or chemical methods, which is useful for a rapid glycan profiling analysis, but lost the information of carrier protein. Another strategy is the analysis of intact glycopeptides after proteolytic digestion. As it provides information on both glycan composition and the attached protein, it is attracting attention in recent years. However, due to the complexity of glycans, interpreting MS output, in terms of glycan structures, attachment sites and glycopeptide is still challenging. Fortunately, recent advances in various searching softwares and tools greatly increased high throughput glycomic and glycoproteomic analysis. In addition, many notable reviews have summarized the informatics softwares and tools for glycan analysis [47,48] and intact glycopeptide analysis [49]. In this section, we introduce some representative software tools for glycan and intact *N*-glycopeptide analysis, as well as newly developed tools for intact *O*-glycopeptide.

### 6.1. Software Tools for Glycan Analysis

In order to determine the possible glycan structure based on experimentally determined masses, firstly we must know the theoretic MS fragments. GlycoWorkbench (Division of Molecular Biosciences, School of Life Sciences, Imperial College London, London, UK. Download link: https://glycoworkbench.software.informer.com/2.1/) is such a tool designed to facilitate manual annotation of mass spectrometry data by matching experimental MS/MS peak lists against theoretical fragments [78]. A particular feature of this tool is that it provides a user-friendly interface for drawing various glycans structure. After selection of fragmentation type and user-defined annotation options, the MS data can be automatically interpreted in several minutes or hours, which greatly accelerates the glycomics study. Currently, Glycoworkbench has become the most widely used software tool in MS-based glycomic studies.

Another software tool for interpretation of glycan profiling from LC/MS data, GlycReSoft (Program for Bioinformatics, Boston University, Boston MA, USA. Download link: http://www.bumc.bu.edu/msr/glycresoft/), has been updated recently. GlycReSoft is a software package implementing supervised and unsupervised scoring methods to enable assignment of peaks to both known and unknown glycan compositions [79]. In the updated version, they developed an optimized algorithm by using network Laplacian regularization to smooth LC-MS assignments of glycan compositions across multiple experimental protocols and thus improve the sensitivity and specificity of glycan composition assignment for LC-MS based experiments [50].

### 6.2. Software Tools for Intact N-Glycopeptide Analysis

In the last decade, the development of a number of bioinformatic tools for glycopeptide identification have facilitated the increase in the *N*-glycoproteome coverage. GPQuest (Center for Biomarker Discovery & Translation, the Johns Hopkins School of Medicine, Baltimore, MA, USA. Download link: https://www.biomarkercenter.org/gpquest) is one of the representative tools for large-scale identification of *N*-glycopeptides. In this algorithm, a spectral library of glycosite-containing peptides in the sample was built. By comparing with the relevant precursor ion of the intact glycopeptides, GPQuest assigns each intact glycopeptide MS/MS spectrum to a specific glycosite-containing peptide [80]. Combined this algorithm with other analytical methods, thousands of *N*-glycopeptides have been identified in different cell lines [51,81].

pGlyco (Institute of Computing Technology, Chinese Academy of Sciences, Beijing, China. Download link: http://pfind.ict.ac.cn/software/pGlyco/index.html) is another representative tool for global characterization of intact *N*-glycopeptides developed in recent years. pGlyco [82] and pGlyco 2.0 [83] use the optimized stepped-energy higher-energy collision dissociation (HCD) to select the mass spectra of glycopeptides and analyze the peptide sequence, and use collision-induced dissociation (CID) to analyze the glycan structure. It is noteworthy that although quality control has been considered in some other bioinformatic tools, pGlyco includes comprehensive quality control when matching mass spectra to glycans, peptides, and glycopeptides. Therefore, pGlyco and pGlyco 2.0 provide accurate identification about glycopeptides with a relatively lower false discovery rate compared to other bioinformatic tools such as Byonic^TM^, the most commonly used commercial software. Using this search engine pGlyco 2.0, more than ten thousand intact *N*-glycopeptides have been identified in five mouse tissues [83], which achieved the deepest and largest scale ever reported [49].

### 6.3. Software Tools for Intact O-Glycopeptide Analysis

Compared to the mature and generic software tools for *N*-glycopeptide, the development of software tools for intact *O*-glycopeptide lags behind. In recent two years, several strategies and interpretation tools for *O*-glycoepeptides are emerging [52,53,54,84]. O-*O*-Search is a new search scheme for the interpretation of *O*-glycopeptide HCD spectra [52]. By setting variable mass tags on the peptide level instead of Ser/Thr residues level, the search space is significantly reduced and many heterogeneous glycan structures could be considered. Currently, this method has been proven to significantly outperform the conventional searching scheme for identification of *O*-glycopeptide in serum.

AOGP is a newly developed bioinformatic tool for intact *O*-glycopeptide analysis on single proteins [84]. By utilizing de novo sequencing for *O*-glycans, a database search strategy for peptide backbones and a false discovery rate (FDR) validation, AOGP automatically interprets intact *O*-glycopeptide mass spectra and provide information on both the glycan and possible glycosylated sites. Currently, AOGP has been found to well exhibit superior performance in identifying intact *O*-glycopeptides of model *O*-glycoproteins. Further improvement and optimization are needed for complex samples.

Overall, these intact glycopeptide identification methods could elucidate the glycan structures and the glycosylation sites precisely. However, there are still several disadvantages. First, the throughput of intact glycoprotein identification is relatively lower than released glycans. Second, the mapping of mass spectrums to glycan structures largely relies on the existing glycan structures in databases. It is still difficult to identify novel glycan structures at large scale. Until recently, a GlycoNovoDB tool provides a de novo sequencing approach to identify glycopeptides carrying novel *N*-glycans [85]. Third, these methods are widely used to identify *N*-glycopeptides but less often used for *O*-glycopeptides. Therefore, more high-throughput methods which can identify novel glycans and *O*-glycopeptides still need to be developed.

## 7. The Latest Integrated Glycoscience Portal

To understand the biological role of glycosylation, we need a comprehensive view of multiple pieces of glycobiology information including glycan structures, glycoprotein sequences and glycosylation sites, interactions between glycan and glycan binding proteins, and relative pathways. Although many resources are technically available, it still takes glycobiologists and researchers a lot of efforts to extract their needed information from different resources. Therefore, integrated portals where all glycoscience-related resources can be accessed from a single website are required. In this section, we introduce three representative integrated glycoscience portals which will benefit researchers in the glycoscience field.

### 7.1. Glycomics@ExPASy

Glycomics@ExPASy (https://www.expasy.org/glycomics) is the glycomics tab of ExPASy, centralizing glycoinformatic resources developed by glycoscientists. The aim of this database is to promote bioinformatics research in glycoscience [19]. It contains comprehensive databases and tools developed and maintained by SIB (such as GlyConnect, SugarBind and UniCarb-DB databases) and external resources (such as CAZy, CSDB, EPS-DB and Glyco3D databases).

**Figure 2:**
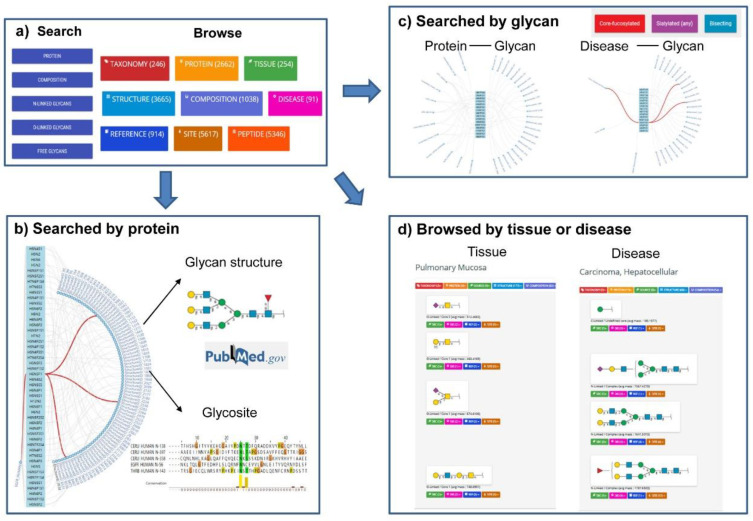
Search interfaces and integrated results of GlyConnect. (**a**) The two interfaces available in GlyConnect: Search and Browse. (**b**) The octopus result of glycan structures and relevant glycosites generated by querying protein UniProt accession (e.g., P00533). (**c**) The octopus results generated by querying glycan structures (e.g., “core-fucosylated”, “sialylated (any)”, and “bisecting”). The associated proteins with these glycans, and disease related to these glycans were shown. (**d**) The Browse interface displays the results of glycan structures related to a certain tissue (e.g., “pulmonary mucosa”) or disease (e.g., “carcinoma, hepatocellular”) in lists of items and proposes a global view of the data.

GlyConnect (https://glyconnect.expasy.org/) is an integrated glycodata platform of Glycomics@ExPASy, which helps characterize the molecular components of protein glycosylation [86]. User can easily search the database by protein names (Figure 2b), monosaccharide compositions, structure properties of glycans (Figure 2c), and free glycans or browse by protein, structure, tissue, disease or cell lines (Figure 2d). A particular feature of this platform is that the results are presented in the form of octopus, which is very easy to understand the relation between protein, glycan, tissue and disease (Figure 2).

### 7.2. Glygen

**Figure 3:**
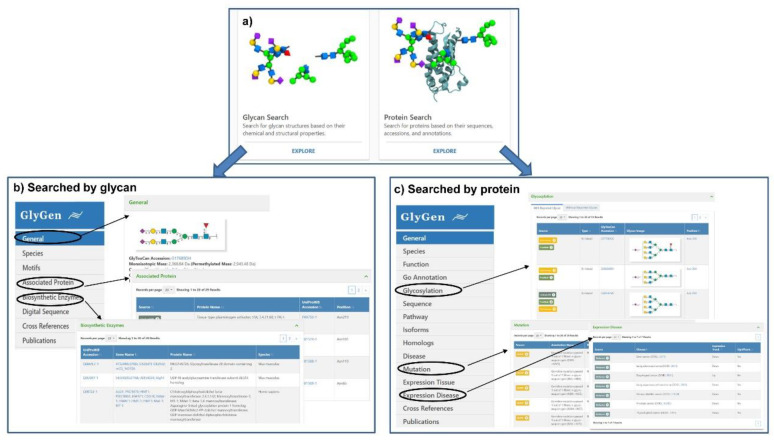
Search interfaces and integrated results of GlyGen. (**a**) The two interfaces available in GlyCen: Glycan Search and Protein Search. (**b**) The integrated result interface when searching with glycan accession (e.g., G17689DH) was shown. The detailed results of general information, associated protein, and biosynthetic enzyme were displayed. (**c**) The integrated result interface when searching with protein accession (e.g., P14210) was shown. The detailed results of glycosylation, mutation, and expression disease were displayed.

GlyGen (https://glygen.org/) is another data integration and dissemination project for carbohydrate and glycoconjugate related data [18]. In the integration process, data are firstly retrieved and extracted from multiple international data sources including the National Center for Biotechnology Information (NCBI), UniProt, the Protein Data Bank (PDB), UniCarbKB, and the GlyTouCan glycan structure repository, and then standardized and harmonized [87]. The current version of the GlyGen Portal provides relevant data about *N*- and *O*-glycans from human, mice, rats, hepatitis C virus, SARS-CoV-1, and SARS-CoV-2. Users can simply search by protein accession, sequences, glycan structure or monosaccharide composition, and then diverse information relevant to glycosylation would be displayed due to the powerful integrated ability of this portal. When searching for a glycan, the information including motifs, associated protein, biosynthetic enzymes and reference will be displayed (Figure 3b). And the information such as function, GO annotation, glycosylation including the structure and glycosite, pathway, disease, mutation, expression, and reference will be present for a protein (Figure 3c). More importantly, the data integrated in the GlyGen project are publicly available in standard formats supported by NCBI and EMBL-EBI, which greatly promotes standardization and sharing of data within the glycomics community.

### 7.3. GlyCosmos

**Figure 4:**
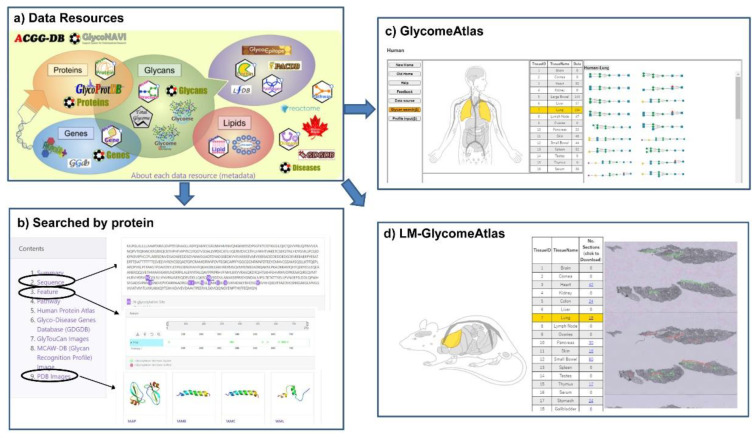
Data resources and integrated results in GlyCosmos. (**a**) An overview of the data resources available in GlyCosmos. Users simply click on the icon for a dataset of interest to access it. (**b**) The integrated result interface when searching with protein accession (e.g., P05067) was shown. The detailed results of sequence and feature with glycosites, and PDB images were displayed. The result interface of GlycomeAtlas (**c**) and LM-GlycomeAtlas (**d**) were shown.

The GlyCosmos Glycoscience Portal (https://glycosmos.org) is the latest integrated web resource for accessing various kinds of glycoscience data resources including glycan-related genes, proteins, lipids, glycomes, pathways and diseases [20]. GlyCosmos integrates multiple resources including the databases developed by JCGGDB and updates with a four-month release cycle. Currently, GlyCosmos Glycogenes has integrated the glycan-related genes from GDGDB, CAZy, FlyGlycoDB, Lipid Maps and KEGG Orthology, while Glycoproteins has integrated glycoproteins from GlycoProtDB and UniProt with relevant links to Reactome pathways, Protein Data Bank, and the Human Proteome Atlas for further functional annotation of each glycoprotein. These data resources are listed on the main page of GlyCosmos (Figure 4a); users can simply click on the icon for a dataset of interest to access it. Also, user can search interest protein by name or accession, then the information on sequence, feature, related pathway, disease and PDB images if available will be displayed (Figure 4b). Compared to GlyConnect and Glygen, the glycoprotein data including their glycosylation sites and the binding lectins which identified by IGOT-MS and lectin microarray experiments are unique to GlyCosmos. Another feature of GlyCosmos is that it provides visualization of glycome profiling data on human, mouse and zebrafish tissue samples (GlycomeAtlas, Figure 4c), and also a lectin microarray-based glycome analysis of mouse tissue (LM-GlycomeAtlas, Figure 4d).

In addition, GlyCosmos also provides three available repositories for data submission. GlyTouCan assigns unique accession codes to all unique glycans, while GlycoPOST and UniCarb-DR archives raw data generated from mass MS experiments on glycans and glycoproteins.

## 8. Discussion and Conclusions

**Figure 5:**
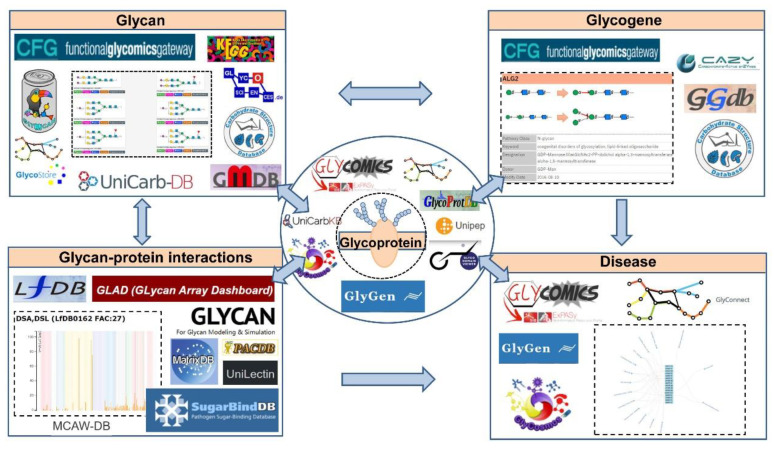
Illustration of integration of various databases to promote functional glycobiology study.

With the development of glycobiology, the association between glycans, glycoproteins, and diseases has been disclosed gradually. For instance, in infectious diseases, glycosylation regulates host–virus interactions, viral immune evasion, and viral release for a range of pathogens such as SARS-CoV, influenza, HIV, and EBOV [88]. In the field of cancer, tumor cells usually bear a specific glycan expression pattern, including truncated *O*-glycans, branched *N*-glycans, and diverse fucosylated and sialylated terminal structures [89]. Based on these facts, glycoproteins have become the promising candidates of disease markers and therapeutic targets, such as in liver fibrosis [90,91], cholangiocarcinoma [92], and lung disease [93]. Thus, fully integrated databases collecting the data on glycan-related molecules, glycogenes and their biological functions are needed. In past decades, with the development of various analytical techniques in glycomics and glycoproteomics, many databases with huge information are emerging. Recently, many notable reviews have summarized these databases and informatics tools [24,48,49,94]; however, most of them are from the perspective of analytical methods and data interpretation, which requires readers to have a certain background in glycomics. In this study, we reviewed the related databases of glycan, glycogenes, and interacting proteins in the process of glycoprotein formation (Figure 5, and Table 1), and gave a brief introduction of representative software tools for glycopeptide (Table 2 and Table 3). We provided an overview of the main features, functionalities, and how to use these representative databases. We believe that this may make it easier for glycobiologists without glycomics or glycoproteomics background or researchers in other fields to use these databases for functional study or interdisciplinary study, ultimately promoting the clinical application of glycobiology.

The integration is an important direction in the development of glycobiology database. The first level of integration should facilitate the cross-reference between databases. Presently, glycan structures are described in different formats including International Union of Pure and Applied Chemistry (IUPAC), LinearCode (r), KEGG Chemical Function (KCF), GlycoCT, GLYDE-II, Oxford, and LINUCS. Therefore, a universal nomenclature for glycan structures is needed for standardization and cross-referencing between databases. For example, the GlycanBuilder, which is developed for drawing glycan structures, also supports the conversion of various glycan structure formats. This tool is included in UniCarbKB and GlyTouCan, and facilitates the collection of all identified glycans in one database.

The second level of integration should link the data of glycan structure and glycoprotein. Due to the limitations of technology, glycan structures and glycosylation sites were identified separately in earlier research. However, only protein-specific glycosylation reveals the exact biological function. As the intact glycopeptide identification technologies are developing rapidly, the glycan information is integrated with glycoprotein data and leads to the establishment of comprehensive glycoprotein databases. In a future glycobiology database, the glycosylated protein will be annotated with the type of glycosylation with all the glycosites mapped. On one hand, all the known glycan structures linked to each glycosylation site will be listed with detailed information. On the other hand, each known glycan structure will be assigned a unique ID, and all the proteins carrying this glycan could be provided.

The third level of integration would collect the information of glycosylated molecules, GBPs, glycogenes, their biological functions and relationship to diseases, and finally establish a comprehensive knowledge database. In the early days, some databases have made efforts in this integration. For example, JCGGDB has integrated MS data of glycans and glycoproteins, lectin affinity data, and glycogene information. KEGG glycan has summarized the glycan structure and their biosynthesis pathways in maps linked to metabolic and signaling pathways, while UniCarbKB has connected the glycan data to protein data in UniProtKB [117]. In recent years, with the development of several international cooperative initiatives, some new diseases with stronger integration abilities have been established, such as Glycomics@ExPASy, GlyGen, and GlyCosmos. These databases establish the standard format of glycans, and attempt to integrate glycoscience data with proteomics and genomics. On this basis, future databases would further strengthen the cross-linking of glycobiology resources with other omics including genomic, proteomic, and metabolomic data, and also include the biological function annotations of glycans and glycoproteins in diseases. Such a comprehensive glycomics database still needs plenty of additional experimental data and bioinformatic analysis. As the glycoscience community has prompted several international cooperative initiatives, this vision will become a reality in the near future.

## Figures and Tables

**Table 1:** Summary of representative glycoinformatic databases.

Name	Description	Retrievable by	URL
**Glycan structure databases**			
CFG glycan structure database [14]	Database providing structural and chemical information on thousands of glycans, including both synthetic glycans and glycans for mammalian species.	Searched by glycan names, composition, molecular weight, motifs, cell lines or tissue samples.	http://www.functionalglycomics.org/glycomics/molecule/jsp/carbohydrate/carbMoleculeHome.jsp
JCGGDB Glycan Mass spectral DataBase [16]	Database containing multi-stage tandem mass spectral of structurally defined *N*-and *O*-linked glycans, and glycolipid glycans.	Searched by glycan composition or m/z value of precursor ion.	https://jcggdb.jp/rcmg/glycodb/Ms_ResultSearch
UniCarbKB [25,26,27]	A curated database of information on glycan structures of glycoproteins, with descriptions of its biological source, supporting reference and experimental methods.	Searched by monosaccharide composition, attached protein, taxonomy or tissue by using an auto completion feature.	http://unicarbkb.org/
KEGG glycan [13]	Database providing information on experimentally determined glycan structures and their metabolic pathways.	Searched by the G number for each glycan structure.	http://www.genome.jp/kegg/glycan/
GLYCOSCIENCE.de [12,33]	An integrated portal containing databases and tools mainly glycan 3D structure analysis.	Searched by monosaccharide composition, molecular formula, structure classification and motifs, as well as NMR atoms or peaks.	http://www.glycosciences.de/
Glycosciences.DB [35]	The main glycan structure database of GLYCOSCIENCE.de, providing published data on glycan structures, their taxonomy, MS and NMR-experimental data, 3D structure models as well as references to PDB entries.	Searched by glycan (sub-)structure, monosaccharide composition, molecular formula, structure classification and motifs, as well as NMR, MS, PDB query, or bibliography queries.	http://www.glycosciences.de/database/
UniCarb-DB [36]	Database providing LC-MS/MS data of glycan structures.	Searched by taxonomy, tissue, reference, mass, composition or precursor mass.	https://unicarb-db.expasy.org/
GlyTouCan [37]	A international glycan sequence repository with a globally unique accession number assigned to each structure.	Searched by text input, motif, or drawing glycan structures in GlycanBuilder. Registered users can additionally register new glycan structures to obtain unique IDs for each structure.	https://glytoucan.org/
GlycoStore [41]	A curated database of information on glycan retention properties with chromatographic, electrophoretic and mass-spectrometry composition data.	Searched by experimental values (GU, AU or time), monosaccharide composition or metadata labels (taxonomy, sample name and the Oxford linear notation).	https://www.glycostore.org
CSDB [42]	Database on the structures of glycans and glycoconjugates in prokaryotes, plants and fungi.	Searched by CSDB ID, glycan substructure, composition, taxonomy, bibliography, NMR signals, conformation ID or GT name.	http://csdb.glycoscience.ru/database/
**Glycoprotein databases**			
GlycoProtDB [56]	Database providing information on *N*-glycoproteins and their glycosylated site(s) identified from *C. elegans*, mouse tissues and human.	Searched by gene ID, gene name, and its description (protein name).	https://acgg.asia/db/gpdb2/
UniPep [59]	Database providing information on *N*-glycopeptides identified from human plasma and tissues including bladder, breast, liver, lymphocytes, cerebrospinal fluid, and prostate.	Searched by gene name, gene symbol, Swiss Prot ID, IPI ID, protein sequence or peptide mass.	http://www.unipep.org/
*N*-GlycositeAtlas [22]	Database providing information on *N*-glycopeptides identified from over 100 publications and unpublished datasets	Searched by gene/protein name, accession number, glycosylation site location, glycosite containing peptide, tissue/liquid/cell line, or publication	http://nglycositeatlas.biomarkercenter.org
GlycoDomain Viewer [95]	Database of *O*-GalNAc proteinsidentified by SimpleCell technology from human and animal cell lines, associated with the verified and predicted glycosylated sites of *N*-glycan, *O*-GalNAc, *O*-Mannose and *O*-Xylose mapping on the protein sequence	Searched by the NCBI gene name or the Uniprot ID	https://glycodomain.glycomics.ku.dk/
**Glycogene databases**			
CAZy [62]	The largest database for display and analysis of genomic, structural and biochemical information on glyco-enzymes	Searched by enzyme family, protein name, organism name, GeneBank or UniProt accession, or EC number.	http://www.cazy.org/
CAZypedia [63]	A comprehensive encyclopedic of detailed structural, and biochemical information on glyco-enzymes, and relevant reference.	Searched by enzyme name or enzyme ID	http://www.cazypedia.org
GlycoGene DataBase [15]	Database providing information of glycogenes on gene sequences, substrate specificities, homologous genes, EC numbers, tissue distribution, KO mouse as well as external links to various databases.	Searched by gene symbols or designations or selected from the list of glycogenes	https://acgg.asia/ggdb2/
CFG glycosyltransferases database	Database providing information of glycosyltransferase on enzyme name, EC number, organism, relevant CFG data, and other data from public databases (PubMed, KEGG, CAZy, SwissProt, and others).	Providing a graphical interface of different glycans. By clicking a monosaccharide, users are directed to the information of the glycosyltransferase which forms this structure.	http://www.functionalglycomics.org/glycomics/molecule/jsp/glycoEnzyme/geMolecule.jsp
CSDB_GT [96]	A curated database of glycosyltransferases in Arabidopsis thaliana, Escherichia coli and Saccharomyces cerevisiae.	Searched by CSDB ID, glycan structure, composition, taxonomy, bibliography, NMR signals, conformation, or GT activity.	http://csdb.glycoscience.ru/gt.html
**Glycan-protein interaction database**			
Lectin Frontier Database [67]	Database providing quantitative interaction data between various glycan and lectins, as well as basic information such as kingdom, monosaccharide specificity on lectins	Searched by keyword or choose categories among Lectin family, Monosaccharide Specificity, or 3D-fold.	http://acgg.asia/lfdb2/
UniLectin [68]	A interactive database for the classification and curation of lectins (with UniLectin3D module), and the prediction of β-propeller lectins (with PropLec module).	Searched by keywords, kingdom order, historical classification, monosaccharide, associate IUPAC sequence, fold of the binding site, or multiple criteria	https://www.unilectin.eu/
PACDB [69]	Database providing information on the interaction of microbial glycan-binding proteins and glycans with host glycan ligands	Selected from the list of disease, pathogen names, monosaccharides, or glycoepitopes	https://acgg.asia/db/diseases/pacdb
SugarBindDB [70]	A curated database providing information on known glycan structure interacted with pathogenic organisms (bacteria, toxins and viruses) in various disease	Searched by pathogenic agents, ligands, recognizing lectins, affected area, references, diseases or multi-criteria.	https://sugarbind.expasy.org/
GLAD [71]	A web-based tool to visualize, analyze, present, and mine glycan array data.	Input data as tab-delimited text files in the correct format	https://glycotoolkit.com/Tools/GLAD/
MCAW-DB [72]	Database providing information on binding affinity of glycan binding proteins to glycan substructures by multiple alignment analysis of glycan array data	Searched by filtering taxa, protein family, investigator and array version.	https://mcawdb.glycoinfo.org/
GlyMDB [73]	Database enabling users to upload their own microarray data, query binder/non-binder classification, discover glycan-binding motif, compare glycan array sample, and cross-link microarray samples to PDB structures	Searched by protein name, protein sequence or PDB ID, or upload microarray spreadsheet file.	http://www.glycanstructure.org/
MatrixDB [74]	A curated database providing information on interactions between extracellular matrix proteins, proteoglycans and polysaccharides.	Searched by a biomolecule, keyword, author, publication or IMEx identifier.	http://matrixdb.univ-lyon1.fr/
**Latest integrated glycoscience portal**			
Glycomics@ExPASy [19]	The glycomics tab of ExPASy, centralizing web-based glycoinformatics databases and tools resources developed by SIB (such as GlyConnect, SugarBind and UniCarb-DB databases) and other external resources to (such as CAZy, CSDB, GlyTouCan and UniCarbKB) to bridge the glycobiology and protein-oriented bioinformatics resources	Click on the link of interest	https://www.expasy.org/glycomics
GlyConnect [86]	The central platform of the Glycomics@ExPASy, providing interactive diagrams that help the user understand relations between glycans, proteins, tissues, diseases, and taxonomy	Either browsed or searched by protein name, ID, or monosaccharide composition, linkage type	https://glyconnect.expasy.org/
Glygen [18]	A web portal data integration, harmonization and dissemination web portal for integrate data and knowledge from diverse disciplines relevant to glycobiology, carbohydrate and glycoconjugate-related data retrieved from multiple international data sources including UniProtKB, GlyTouCan, UniCarbKB and other key resources.	Searched by protein accession, sequences, glycan structure or monosaccharide composition.	https://glygen.org/
GlyCosmos [20]	An integrated web resource including the database of JCGGDB and providing information on glycan-related genes, proteins, lipids, glycomes, pathways and diseases to integrate the glycosciences with the life sciences	Searched by protein name, protein accession, species, or various glycan search tools, such as by mass, composition, graphical glycan structure or monosaccharide composition.	https://glycosmos.org

**Table 2:** Bioinformatic tools for glycopeptide identification.

Bioinformatic Tools	Glycan Identification Method	Peptide Identification Method
GlycoWorkbench [78]	MS, MS/MS	MS
GlycReSoft [50,79]	LC-MS	LC-MS/MS
Byonic [97]	match by glycan mass	ETD, HCD
Protein Prospector [98]	match by glycan mass	ETD
pGlyco [82,83]	CID	HCD
GlycoNovoDB [85]	HCD	HCD
GPQuest [80]	HCD	HCD
GlycoPeptide Finder (GPFinder) [4]	QTOF-CID	QTOF-CID
MAGIC [99]	QTOF-CID	QTOF-CID
GlycoMaster DB [100]	HCD	ETD
GlycoFinder [101]	low energy HCD	HCD
Sweet-Heart [102]	CID	MS^3^
Sweet-Heart for HCD [103]	CID	HCD
GlycoFragWork [104]	CID	ETD
pMatchGlyco [105]	match by MS/MS spectra	HCD
GlycoPeptideSearch [106,107]	CID	ETD
ArMone [108]	CID	HCD
GlypID 2.0 [109]	CID	HCD
O-*O*-Search [52]	HCD	HCD
AOGP [84]	HCD	HCD

**Table 3:** Bioinformatic tools for glycosylation site prediction.

Bioinformatic Tools	Description	URL
NetNGlyc	*N*-glycosylation site prediction	http://www.cbs.dtu.dk/services/NetNGlyc/
NetOGlyc [110]	*O*-GalNAc site prediction	http://www.cbs.dtu.dk/services/NetOGlyc/
YinOYang [60]	*O*-(beta)-GlcNAc and phosphorylation site prediction	http://www.cbs.dtu.dk/services/YinOYang/
DictyOGlyc [111]	*O*-(alpha)-GlcNAc site prediction	http://www.cbs.dtu.dk/services/DictyOGlyc/
NetCGlyc [112]	C-mannose site prediction	http://www.cbs.dtu.dk/services/NetCGlyc/
Big-PI Predictor [113]	GPI-anchor prediction	http://mendel.imp.ac.at/sat/gpi/gpi_server.html
GPI-SOM [114]	GPI-anchor prediction	http://genomics.unibe.ch/cgi-bin/gpi.cgi
PredGPI [115]	GPI-anchor prediction	http://gpcr.biocomp.unibo.it/predgpi/pred.htm
FragAnchor [116]	GPI-anchor prediction	http://navet.ics.hawaii.edu/~fraganchor/NNHMM/NNHMM.html.
